# Integrative analysis of transcriptomic and metabolomic profiles reveals enhanced arginine metabolism in androgen-independent prostate cancer cells

**DOI:** 10.1186/s12885-023-11707-3

**Published:** 2023-12-16

**Authors:** Xingchen Dai, Xinyi Shi, Mingxiu Luo, Pu Li, Yujing Gao

**Affiliations:** 1 Key Laboratory of Fertility Preservation and Maintenance of Ministry of Education, Department of Biochemistry and Molecular Biology, School of Basic Medical Sciences, Ningxia Medical University, Yinchuan, China; 2 Department of Nephrology, First Affiliated Hospital of Fujian Medical University, Fuzhou, China; 3 Ankang Central Hospital, Ankang, China; 4 Department of Pediatrics, Ruijin Hospital, Shanghai Jiao Tong University School of Medicine, Shanghai, China; 5 National Health Commission Key Laboratory of Metabolic Cardiovascular Diseases Research, Ningxia Medical University, Yinchuan, China

**Keywords:** Transcriptomics, Metabolomics, LNCaP cells, CRPC

## Abstract

**Background:**

Prostate cancer is a common solid tumor that affects a significant number of men worldwide. Conventional androgen deprivation therapy (ADT) increases the risk of developing castration-resistant prostate cancer (CRPC). Effective clinical management of patients with CRPC is challenging due to the limited understanding.

**Methods:**

In this study, transcriptomic and metabolomic profiles of androgen-dependent prostate cancer cell line LNCaP and the androgen-independent cells developed from LNCaP cells (LNCaP-ADR) were investigated using RNA-sequencing and LC-MS/MS, respectively. The differentially expressed genes and metabolites were analyzed, and integrative analysis of transcriptomic and metabolomic data was further conducted to obtain a comprehensive understanding of the metabolic characteristics in LNCaP-ADR cells. Quantitative real-time PCR (QPCR) was employed to ascertain the mRNA expression levels of the selected differentially expressed genes.

**Results:**

The arginine and proline metabolism pathway was identified as a commonly altered pathway at both the transcriptional and metabolic levels. In the LNCaP-ADR cells, significant upregulation was observed for metabolites including 5-Aminopentanoic acid, L-Arginine, L-Glutamic acid, N-Acetyl-L-alanine, and Pyrrole-2-carboxylic acid at the metabolic level. At the transcriptional level, *MAOA*, *ALDH3A2*, *ALDH2*, *ARG1*, *CKMT2*, and *CNDP1* were found to be significantly upregulated in the LNCaP-ADR cells. Gene set enrichment analysis (GSEA) identified various enriched gene sets in the LNCaP-ADR cells, encompassing inflammatory response, 9plus2 motile cilium, motile cilium, ciliary plasm, cilium or flagellum-dependent cell motility, cilium movement, cilium, response to endoplasmic reticulum stress, PTEN DN.V1 DN, SRC UP.V1 UP, IL15 UP.V1 DN, RB DN.V1 DN, AKT UP MTOR DN.V1 UP, VEGF A UP.V1 UP, and KRAS.LUNG.BREAST UP.V1 UP.

**Conclusions:**

These findings highlight the substantial association between the arginine and proline metabolism pathway and CRPC, emphasizing the need to prioritize strategies that target dysregulated metabolites and differentially expressed genes as essential interventions in the clinical management of CRPC.

## Introduction

 Prostate cancer (PCa) is the second most prevalent solid tumor in men worldwide and ranks as the fifth leading cause of cancer-related deaths [1]. Annually, around 1.3 million new cases of prostate cancer are diagnosed. Approximately 10 million men are currently living with prostate cancer, and among them, approximately 700,000 have metastatic disease. Metastatic prostate cancer claims the lives of over 400,000 individuals annually, and it is projected to more than double by 2040 [2]. The occurrence of prostate cancer is primarily influenced by factors such as age, family history, ethnicity, and genetic susceptibility [3]. Androgen deprivation therapy (ADT), achieved through medical or surgical castration, has been the standard treatment for metastatic prostate cancer for several decades. However, resistance to castration eventually develops, leading to the progression of castration-resistant prostate cancer (CRPC), which can progress rapidly and result in a life expectancy of 2 to 4 years [4, 5]. The development of castration-resistant prostate cancer (CRPC) poses a significant challenge in prostate cancer treatment. Despite extensive research and reports on CRPC, the underlying mechanism of its formation remains incompletely understood. Currently, there are no specific and reliable diagnostic methods available to distinguish CRPC from typical prostate cancer in the early stages.

Transcriptomics allows us to interpret functional elements of the genome and reveal global gene expression profiles associated with disease. Based on gene expression data, researchers have uncovered pathway dysregulation and transcriptional programs associated with PCa progression and metastasis [6], which helps to predict the prognosis of the patients [7], and understand the mechanism for the process of tumorigenesis [8]. Metabolomics is currently the main method for early diagnosis and precision medicine, focusing on small molecules to reveal changes in the metabolism of biological systems [9]. Metabolomics uncovers differences in metabolite concentrations or alterations in metabolic pathways [10], which can provide insights into cancer progression from a metabolic perspective [11], promising to be a valuable tool for the early detection of PCa and consequently leading to earlier disease treatment and clinical outcomes improvement [12]. Moreover, the integration of metabolomic and transcriptomic data may provide greater insight into the disease than either approach alone. Particularly, dysregulation of metabolites and genes in the same biological process will strengthen the potential implication of the process in the disease.

In the present study, we conducted RNA-sequencing and metabolomics analyses on the androgen-dependent prostate cancer cell line LNCaP and the androgen-independent cells generated from LNCaP. By separate and integrated analyses of transcriptomic and metabolomic profiles of the two cell lines, we summarized the specific transcriptomic and metabolomic features of LNCaP-ADR cells that different from LNCaP parental cells. Our study will not only help to decipher the mechanism underlying CRPC progression, but also provide potential biomarkers for the risk assessment of CRPC.

## Materials and methods

### Cell culture

LNCaP parental cells were purchased from American Type Culture Collection (ATCC) and preserved in our laboratory. Cells were cultured in RPMI 1640 medium supplemented with 10% fetal bovine serum (FBS), 100 μg/mL streptomycin, and 100 U/mL penicillin (Beyotime, Cat. No. ST488). The cells were incubated at 37℃ in a 5% CO_2_ environment. An androgen-independent prostate cancer cell line, LNCaP-ADR, was generated by culturing the LNCaP parental cells under androgen-deprivation conditions. The cells were maintained in phenol red-free RPMI 1640 medium (Gibco, Cat. No. 11835030) supplemented with 10% charcoal-dextran stripped fetal bovine serum (cFBS; Biological Industries, Cat. No. 04-201-1A), streptomycin, and penicillin.

### RNA-sequencing

Total RNA of LNCaP parental cells (L group) and LNCaP-ADR cells (LA group) was isolated and purified using TRIzol reagent (Invitrogen, Carlsbad, CA, USA) following the manufacturer’s procedure. The RNA amount and purity of each sample was quantified using NanoDrop ND-1000 (NanoDrop, Wilmington, DE, USA). The RNA integrity was assessed by Bioanalyzer 2100 (Agilent, CA, USA) with RIN number > 7.0, and confirmed by electrophoresis with denaturing agarose gel. The RNA-Sequencing analysis was conducted by LC-Bio Technologies Co., Ltd (Hangzhou, China).

### Gene Ontology (GO) and Kyoto Encyclopedia of genes and genomes (KEGG) enrichment analysis

For functional enrichment analysis, all differentially expressed genes (DEGs) were annotated to terms in the GO databases and significantly enriched GO terms were identified among the DEGs using a *p*-value threshold of less than 0.05. The GO analysis classified the enriched terms into three subgroups: biological process (BP), cellular component (CC), and molecular function (MF). Additionally, all DEGs were mapped to the KEGG database, and significantly enriched KEGG pathways were identified using a *p*-value threshold of less than 0.05.

### Gene Set Enrichment Analysis (GSEA)

GSEA Java (v4.1.0) was employed for Gene expression enrichment analysis (GSEA) analysis. GSEA analysis was conducted between the LNCaP-ADR cells (LA group) and LNCaP parental cells (L group) using transcriptomic data. The reference gene sets selected for analysis included h.all.v7.4.symbols.gmt (hallmark gene sets), c5.all.v7.4.symbols.gmt (ontology gene sets), and c6.all.v7.4.symbols.gmt (oncogenic signature gene sets). In each analysis, a total of 1,000 gene sets were arranged to determine significantly different pathways. Gene set permutations were performed 1,000 times to identify pathways with significant differences. The enrichment magnitude was quantified using the normalized enrichment score (|NES|xx1), while the statistical significance was assessed using the false discovery rate (FDR<25%). A nominal *p*-value < 5% was chosen as the cut-off criteria for statistical significance.

### Metabolites extraction

A certain amount extract solution (acetonitrile: methanol: water = 2: 2: 1) was added to the cell samples (LNCaP-ADR and LNCaP-parental cell lines respectively, five replicates for each cell line). After 30 s vortex, the samples were freezed and thawed with liquid nitrogen for 3 times. Then the samples were incubated at -40 ℃ for 1 h and centrifuged at 12,000 rpm for 15 min at 4 ℃. 800 µL supernatant was transferred to an EP tube and dried in a vacuum concentrator. Then acetonitrile: methanol: water = 2: 2: 1, with isotopically-labelled internal standard mixture was added in proportion to redissolve. The resulting supernatant was transferred to a fresh glass vial for LC/MS analysis. The quality control (QC) sample was prepared by mixing an equal aliquot of the supernatants from all of the samples.

### LC-MS/MS analysis

LC-MS/MS analyses were performed using an UHPLC system (Vanquish, Thermo Fisher Scientific) with a UPLC BEH Amide column (2.1 mm × 100 mm, 1.7 μm) coupled to Q Exactive HFX mass spectrometer (Orbitrap MS, Thermo). The mobile phase consisted of 25 mmol/L ammonium acetate and 25 ammonia hydroxide in water(pH = 9.75)(A) and acetonitrile (B). The auto-sampler temperature was 4℃, and the injection volume was 3 µL.

The QE HFX mass spectrometer was used for its ability to acquire MS/MS spectra on information-dependent acquisition (IDA) mode in the control of the acquisition software (Xcalibur, Thermo). In this mode, the acquisition software continuously evaluates the full scan MS spectrum. The ESI source conditions were set as following: sheath gas flow rate as 30 Arb, Aux gas flow rate as 25 Arb, capillary temperature 350 ℃, full MS resolution as 60,000, MS/MS resolution as 7500, collision energy as 10/30/60 in NCE mode, spray Voltage as 3.6 kV (positive) or -3.2 kV (negative), respectively. The LC-MS/MS analysis was conducted by BIOTREE Technologies Co., Ltd (Shanghai, China).

### Principal Component Analysis (PCA)

Internal standard normalization method was employed in the data analysis. The final dataset containing the information of peak number, sample name and normalized peak area was imported to SIMCA16.0.2 software package (Sartorius Stedim Data Analytics AB, Umea, Sweden) for multivariate analysis. Data was scaled and logarithmic transformed to minimize the impact of both noise and high variance of the variables. After these transformations, PCA, an unsupervised analysis that reduces the dimension of the data, was carried out to visualize the distribution and the grouping of the samples. 95%confidence interval in the PCA score plot was used as the threshold to identify potential outliers in the dataset.

### Orthogonal Projections to Latent Structures-Discriminant Analysis (OPLS-DA)

SIMCA software (V16.0.2, Sartorius Stedim Data Analytics AB, Umea, Sweden) was utilized for logarithmic (LOG) conversion and UV formatting of the data. Initially, an OPLS-DA modeling analysis was performed on the first principal component to assess the model’s quality using 7-fold cross-validation. The interpretability of the model to the categorical variable Y (R2Y) and the predictability of the model (Q2), obtained after cross-validation, were used to evaluate its validity. Subsequently, a permutation test was conducted by randomly changing the order of the categorical variable Y multiple times to obtain different random Q values. This test further assessed the model’s validity. Based on the data results, an OPLS-DA model was constructed, and scatter plots and permutation test results were obtained.

### Screening of differentially expressed metabolites

Differentially expressed metabolites were screened using the following criteria: *p*-value of Student’s *t*-test < 0.05 and Variable Importance in the Projection (VIP) of the first principal component of the OPLS-DA model > 1. The results of screening differential metabolites were visualized using volcano plots.

### Metabolic pathway analysis of differentially expressed metabolites

The differential metabolites were mapped to authoritative metabolite databases such as KEGG and PubChem to obtain matching information. After that, metabolic pathways in the pathway database specific to the corresponding species (human) were searched and analyzed. Pathway screening criteria were set as follows: FDR < 0.1 and Impact > 0. The key pathway with the highest correlation to the metabolite differences was determined. The analysis was conducted using R version 3.6.1.

### Clustering correlation heatmap and correlation network map

To investigate the relationship and correlation between differentially expressed metabolites and genes, clustering correlation heatmaps and correlation network maps were generated. The clustering correlation heatmap with signs and correlation network were created using the OmicStudio tools at https://www.omicstudio.cn/tool. The correlation calculation (Pearson correlation) was performed using the stats package in R version 3.6.3 (2020-02-29). The analysis was conducted using R version 3.6.3 (2020-02-29) and the igraph package version 1.2.6.

### Quantitative real-time PCR (QPCR)

Total RNA of LNCaP cells were extracted using TRIZOL reagent (Invitrogen, USA), and reversely transcribed to cDNA using an RNA reverse transcription kit (Takara, Japan). QPCR was performed using the ABI 7500 Fast Real-Time PCR system (Applied Biosystems, Carlsbad, CA). Primers were synthesized by Shanghai Sangong Biotechnology Co., Ltd. (China). The Primers for the QPCR are as follows: *MAOA*, forward, 5’-GAATCAAGAGAAGGCGAGTATCG-3’, reverse, 5’-GGCAGCAGATAGTCCTGAAATG-3’; *ARG1*, forward, 5’-GTGGAAACTTGCATGGACAAC-3’, reverse, 5’- AATCCTGGCACATCGGGAATC-3’; *ALDH2*, forward, 5’- ATGGCAAGCCCTATGTCATCT-3’, reverse, 5’- CCGTGGTACTTATCAGCCCA-3’, *ALDA3A2*, forward, 5’- AAACCAGTTAAGAAGAACGTGCT-3’, reverse, 5’- CGAAGGGGTAATTCCAAGCTC-3’; *CKMT2*, forward, 5’- CCAAGCGCAGACTACCCAG-3’, reverse, 5’ GGTGTCACCTTGTTGCGAAG-3’, *CNDP1*, forward, 5’- ATGGTCAGAGTCTTCCAATACCT-3’, reverse, 5’- TAGAAGCACACGGTGCCTTTC-3’; *GAPDH*, forward, 5’- GGACTCATGACCACAGTCCA-3’, reverse, 5’- CCAGTAGAGGCAGGGATGAT-3’. The mRNA levels were normalized using GAPDH as a reference, and the relative expression levels were determined using the 2^−ΔΔCt^ method.

### Statistical analysis

GraphPad Prism version 8.3.0 software (GraphPad Inc., San Diego, CA, USA) was utilized for the analysis of the QPCR data, with the results presented as the mean ± standard deviation (SD). Unpaired t-tests were conducted to compare two groups wherever applicable. A significance threshold of *P* < 0.05 was used to determine statistical significance. Each experiment involved a minimum of three replicates.

## Result

### Identification of differentially expressed genes in LNCaP-ADR cells

**Fig. 1:**
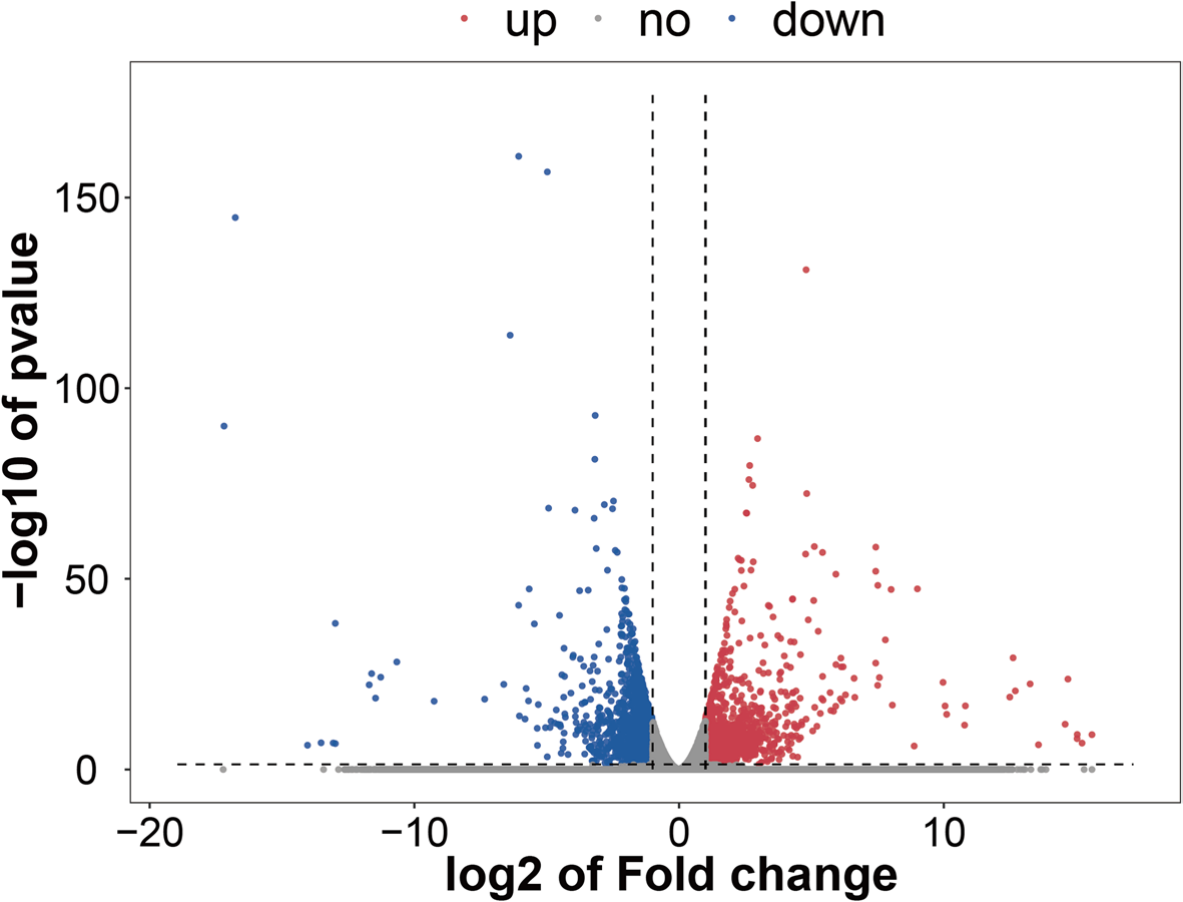
Volcano map of DEGs between LNCaP-ADR cells and LNCaP parental cells. The volcano map displays the differentially expressed genes (DEGs) between LNCaP-ADR cells (LA group) and LNCaP parental cells (L group). The x-axis is presented on a log2 scale, representing the fold change in gene expression between LNCaP-ADR cells and LNCaP parental cells (log2(fold change)). Negative values on the x-axis indicate downregulation, whereas positive values indicate upregulation. The y-axis is presented on a log10 scale, depicting the *p*-values, which determine the significance level of expression difference. Red dots on the volcano map indicate significantly upregulated genes with at least a two-fold change, while blue dots represent significantly downregulated genes, also with at least a two-fold change

**Fig. 2:**
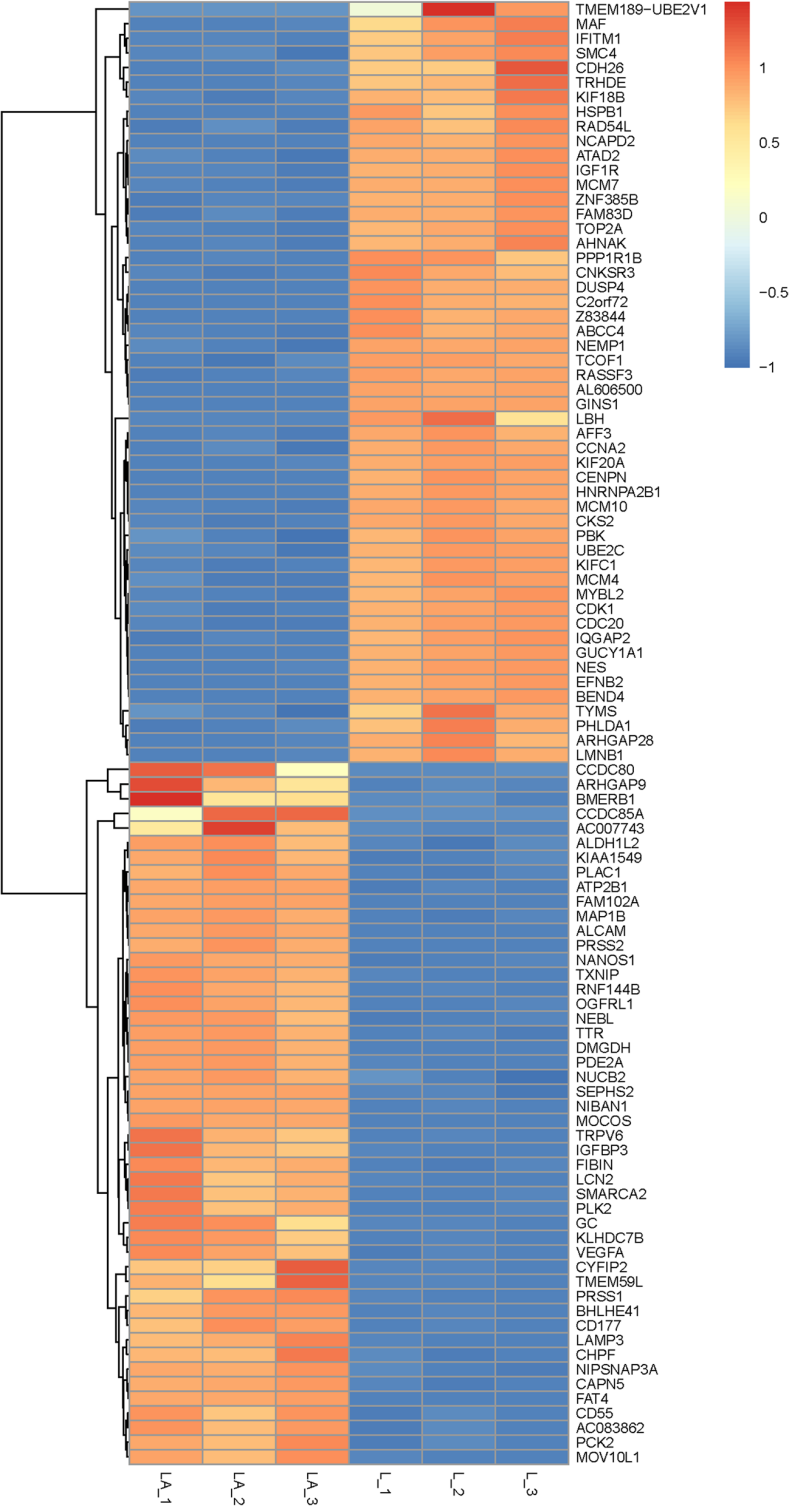
Heatmap depicting the differentially expressed genes between LNCaP-ADR cells and LNCaP parental cells. The heatmap uses red or orange color to indicate high expression, while blue color represents low expression of the genes in LNCaP-ADR cells (LA group) and LNCaP parental cells (L group)

To elucidate the possible genes involved in the development of androgen deprivation resistance, we conducted RNA-sequencing (RNA-seq) of the whole transcriptome of androgen-dependent LNCaP cells (L group) and androgen-independent prostate cancer cells generated from LNCaP cells, referred as to LNCaP-ADR cells (LA group) to identify the changes in gene expression. A total of 60,612 genes were identified. DEG analysis revealed 3,177 genes with significant differential expression (*P* < 0.05). Among these, 1,749 genes showed an up-regulation trend in LNCaP –ADR cells, while 1,428 genes showed a down-regulation trend in the cells (Fig. 1). The top 100 differentially expressed genes were listed as heat map in Fig. 2.

### Highly expressed GO terms in the LNCaP-ADR cells associated with cell replication and DNA maintenance

**Fig. 3:**
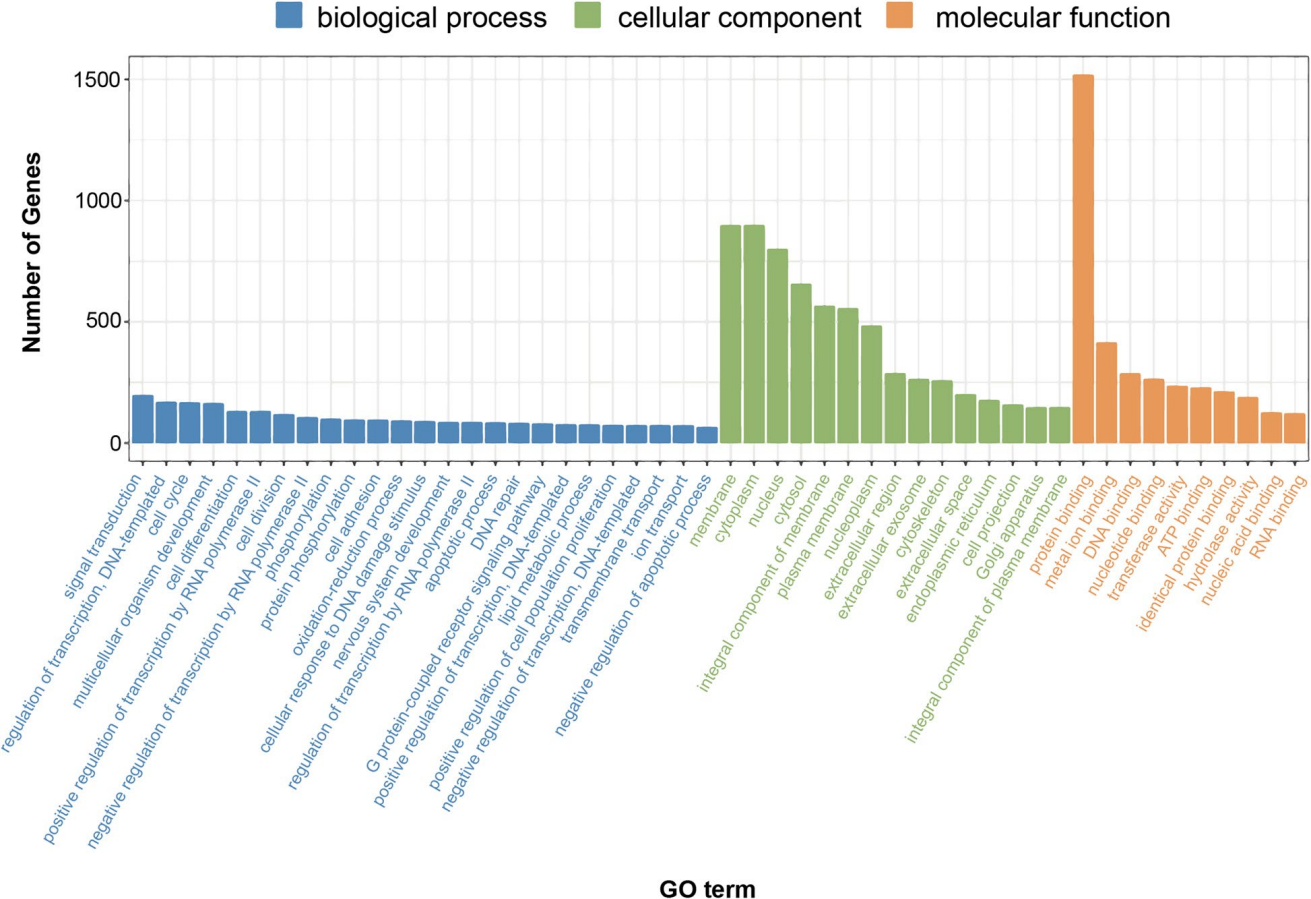
Classification and analysis of GO functions of the DEGs between LNCaP-ADR cells and LNCaP parental cells. The x-axis represents the GO classification, while the y-axis represents the number of genes

A total of 8,335 GO terms were detected, with 723 GO terms found to be significantly enriched among the DEGs (*P* < 0.05). These included 458 GO terms in biological processes, 121 GO terms in cellular components, and 142 GO terms in molecular functions. The top 25, 15 and 10 annotation items in the above three items were displayed in Fig. 3 respectively.

### Significantly enriched pathways in LNCaP-ADR cells

**Fig. 4:**
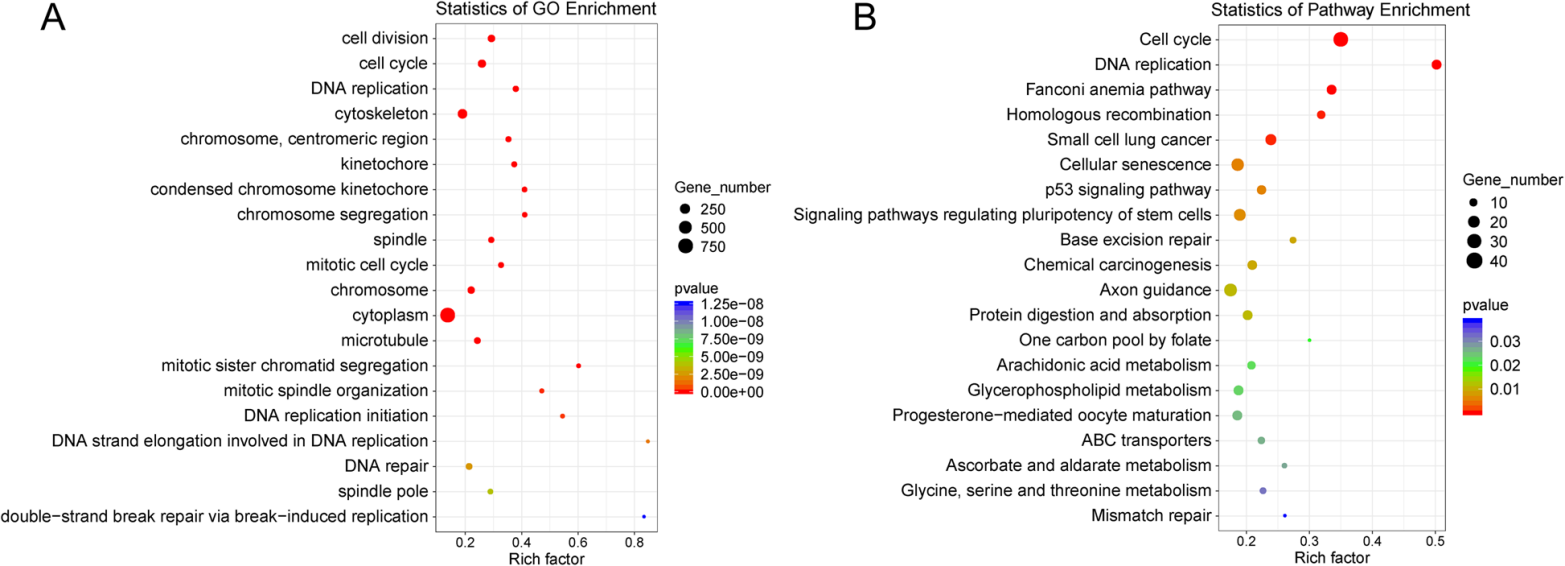
Significant GO terms & KEGG pathways differing in LNCaP-ADR vs. LNCaP parental cells, top 20. **A** The scatter plot represents the top 20 enriched GO terms in LNCaP-ADR cells. **B** The scatter plot represents the top 20 enriched KEGG pathways in LNCaP-ADR cells

Upon comparing the two groups, the top 20 GO terms that were highly expressed in the LNCaP-ADR cells (LA group) were primarily associated with cell replication, mitosis, DNA replication, and DNA repair (Fig. 4A). Compared with the LNCaP parental cells (L group), the top 20 KEGG pathways in LNCaP-ADR cells were mainly related to cell cycle, cell senescence, base repair, amino acid metabolism, lipid metabolism, and so on. (Fig. 4B).

### Differential gene expression analysis reveals enriched gene sets in the LNCaP-ADR cells

**Fig. 5:**
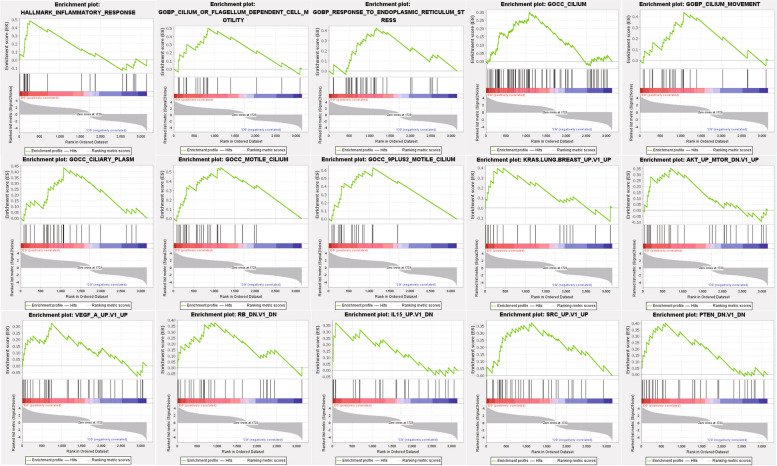
GSEA results across three datasets: Hallmark gene sets, Ontology gene sets, and Oncogenic signature gene sets. The LNCaP-ADR cells showed enrichment in 15 pathways

In the analysis of hallmark gene sets, 14 out of 36 gene sets were upregulated in the LNCaP-ADR cells (LA group). One gene set showed significant enrichment at a false discovery rate (FDR) < 25%, primarily related to inflammatory response (Fig. 5).

In the analysis of ontology gene sets, 507 out of 2,359 gene sets were upregulated in the LNCaP-ADR cells. Seven gene sets were significantly enriched at FDR < 25%, mainly associated with 9 plus 2 motile cilium, motile cilium, ciliary plasm, response to endoplasmic reticulum stress, cilium or flagellum-dependent cell motility, cilium movement, and cilium (Fig. 5).

In the analysis of oncogenic signature gene sets, 54 out of 160 gene sets were upregulated in the LNCaP-ADR cells. Seven gene sets were significantly enriched at FDR < 25%, primarily related to PTEN, SRC, IL15, RB, AKT, VEGF, and KRAS (Fig. 5).

### Metabolite profiling reveals significant changes in LNCaP-ADR cells

**Fig. 6:**
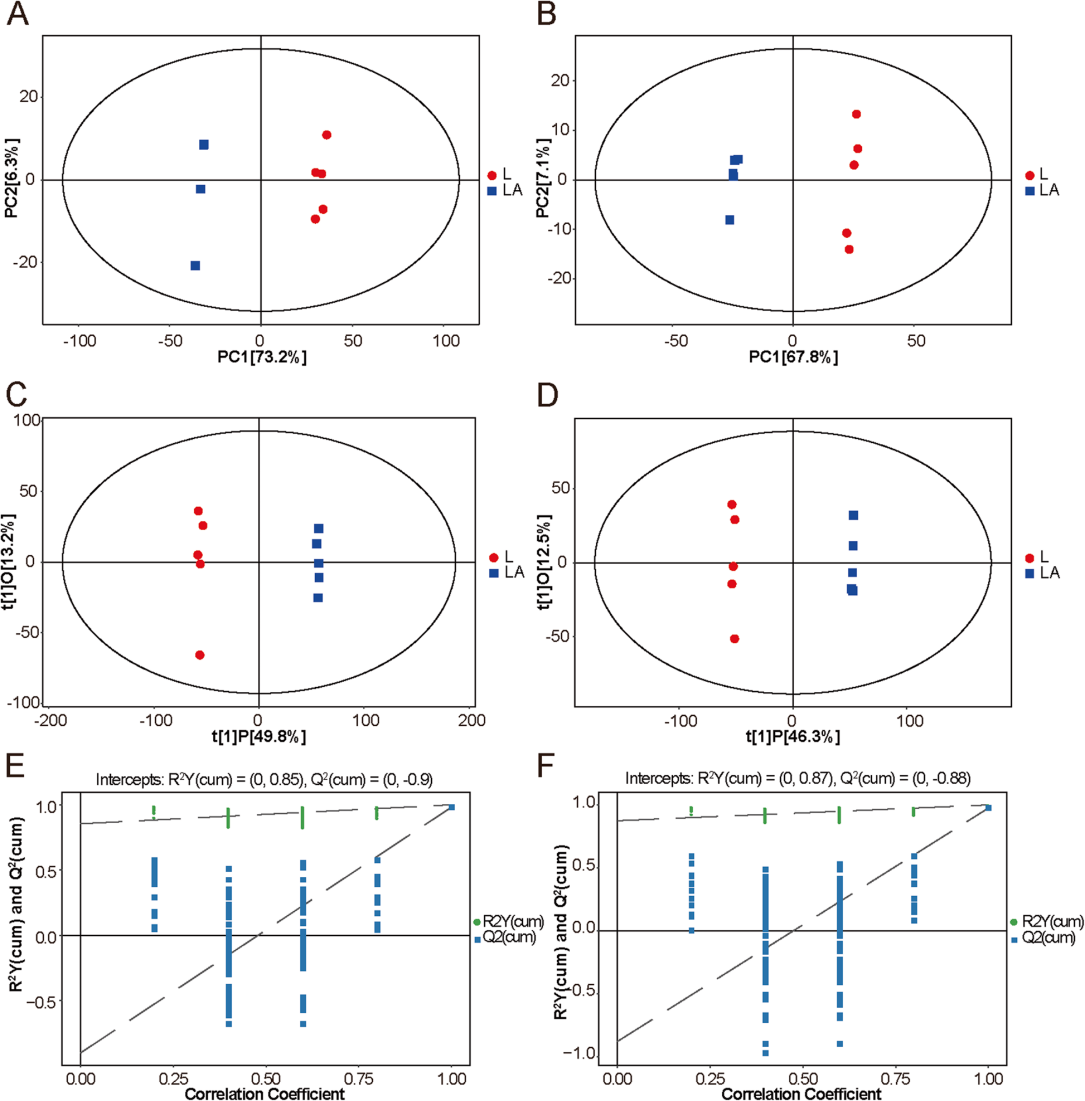
Multivariate analysis of the metabolic profile of LNCaP-ADR cells and LNCaP parental cells. **A**, **B** PCA score plots in the POS model **A** and NEG model (**B)**. **C**, **D** OPLS-DA score plots in the POS model (**C**) and NEG model (**D**). **E**, **F** Permutation tests of the OPLS-DA model in the POS model (**E**) and NEG model (**F**). PCA, principal component analysis. OPLS-DA, orthogonal projections to latent structures- discriminant analysis

**Fig. 7:**
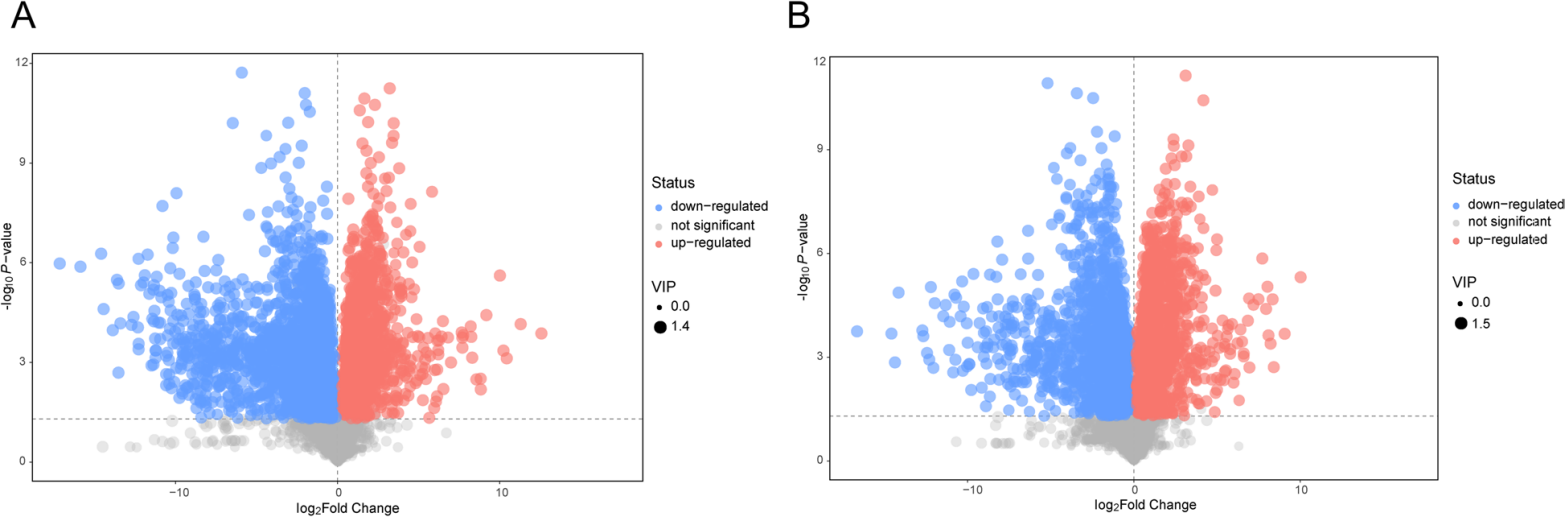
Identification of differential metabolites between LNCaP-ADR cells and LNCaP parental cells. **A**, **B** Volcano plots depicting the differential metabolites in LNCaP-ADR cells compared to LNCaP parental cells, presented separately for the POS model (**A**) and NEG model (**B**). In the plots, red dots indicate upregulated metabolites, blue dots indicate downregulated metabolites, and gray dots represent unaffected metabolites

PCA and OPLS-DA were used to analyze the metabolite data matrix of the LA group and L group samples. The PCA and OPLS-DA score plots demonstrated a clear discrimination between the two groups, indicating significant changes in metabolite composition in LNCaP-ADR cells (LA group) compared to LNCaP parental cells (L group) (Fig. 6A-D). The permutation test of the OPLS-DA model confirmed the robustness of the original model and indicated no overfitting (Fig. 6E, F). The volcano plots showed that 2,243 endogenous differentially expressed metabolites (DEMs) were significantly down-regulated and 1,215 DEMs were significantly up-regulated in the LA group under the positive ion mode; while 1,548 endogenous DEMs were significantly down-regulated and 1,564 DEMs were significantly up-regulated in the LA group under the negative ion mode (*P* < 0.05) (Fig. 7A, B).

### Metabolic pathways enriched in DEMs of LNCaP-ADR cells

All markers of DEMs were functionally annotated and subjected to pathway enrichment analysis using KEGG. Four metabolic pathways that met the screening criteria (*P* < 0.05, FDR < 0.1, Impact > 0) were identified in the positive ion mode: Arginine and proline metabolism, Sphingolipid metabolism, Beta-alanine metabolism, and Glycerophospholipid metabolism. In the negative ion mode, five metabolic pathways that met the screening criteria (*P* < 0.05, FDR < 0.1, Impact > 0) were identified: Arginine and proline metabolism, Alanine, aspartate and glutamate metabolism, Pyrimidine metabolism, Taurine and hypotaurine metabolism, and Purine metabolism (Tables 1 and 2).

**Table 1:** Pathway analysis: altered metabolites in LNCaP-ADR vs. LNCaP parental cells using POS mode

Pathway name	Match status	*p*	-Ln(p)	Holm p	FDR	Impact
Arginine and proline metabolism	12/77	0.00057715	7.4574	0.046172	0.046172	0.42386
Sphingolipid metabolism	6/25	0.0015432	6.4739	0.12191	0.061728	0.33382
Beta-Alanine metabolism	6/28	0.0028776	5.8508	0.22446	0.075303	0.12176
Glycerophospholipid metabolism	7/39	0.0037651	5.582	0.28991	0.075303	0.25106

**Table 2:** Pathway analysis: altered metabolites in LNCaP-ADR vs. LNCaP parental cells using NEG mode

Pathway name	Match status	*p*	-Ln(p)	Holm p	FDR	Impact
Arginine and proline metabolism	12/77	0.000064435	9.6498	0.0051548	0.0051548	0.40538
Alanine, aspartate and glutamate metabolism	6/24	0.00036124	7.926	0.028538	0.01445	0.23362
Pyrimidine metabolism	9/60	0.00075231	7.1924	0.05868	0.020061	0.25617
Taurine and hypotaurine metabolism	5/20	0.0011554	6.7633	0.088965	0.022347	0.42267
Purine metabolism	11/92	0.0013967	6.5737	0.10615	0.022347	0.13774

### Pathway-specific upregulation of metabolites in LNCaP-ADR cells

**Fig. 8:**
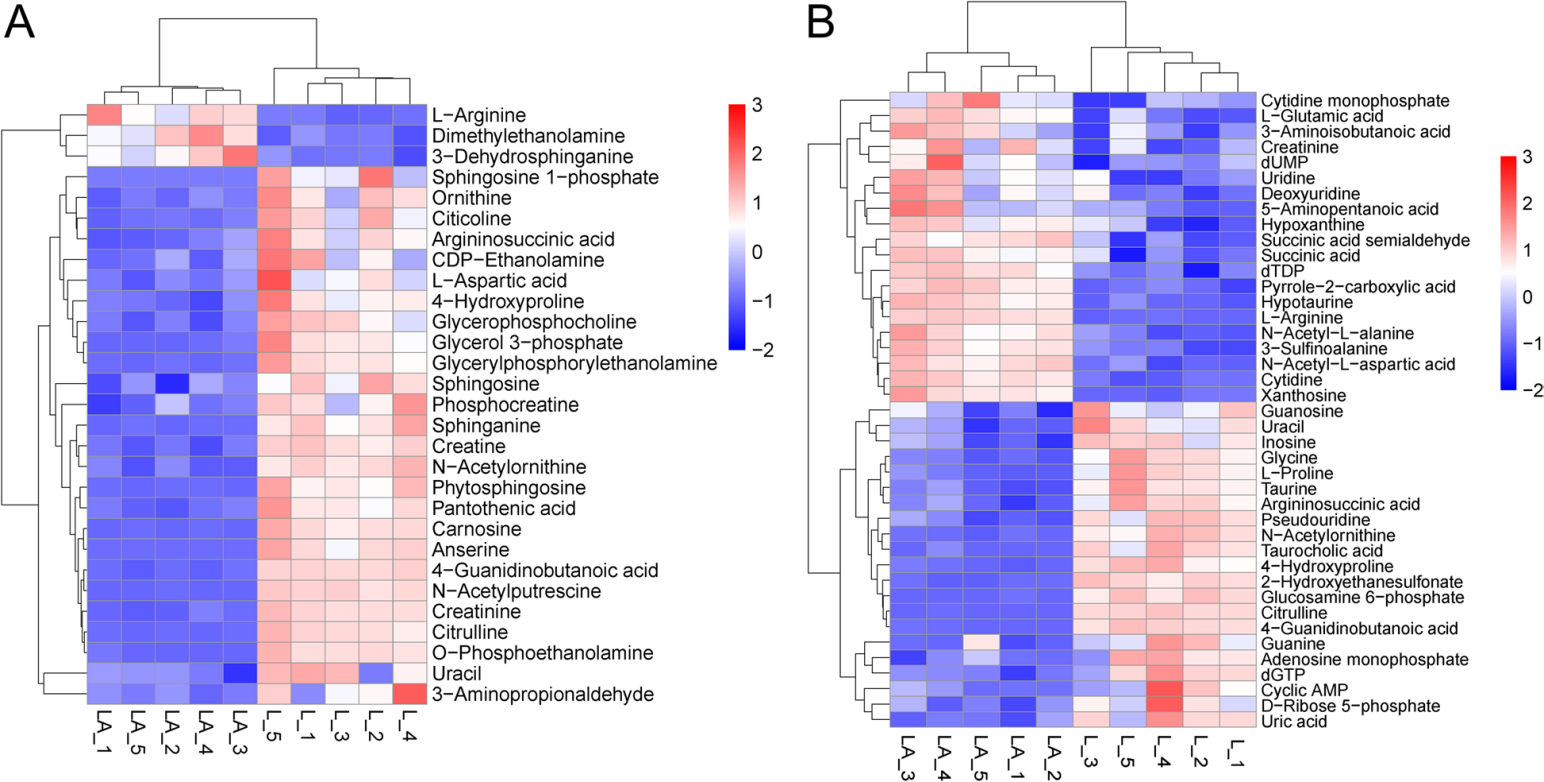
Differential metabolites between LNCaP-ADR cells and LNCaP parental cells. **A** Heatmap illustrating the results of hierarchical clustering analysis comparing LNCaP-ADR cells (LA group) with LNCaP parental cells (L group) in the POS model, and (**B**) in the NEG model. The x-axis represents different experimental groups, the y-axis represents the investigated differential metabolites, and the color represents the relative expression of the corresponding metabolite in the corresponding sample

The DEMs involved in the identified metabolic pathways were subjected to hierarchical clustering analysis. The heat maps showed the up-regulated expression levels of intermediate metabolites and/or end products in glycerophospholipid, sphingomyelin, Beta-Alanine, and arginine and proline metabolism pathways in the LNCaP-ADR cells (LA group) under the positive ion mode (Fig. 8A). Under the negative ion mode, the expression levels of intermediate metabolites and/or end products in arginine and proline metabolism, alanine, aspartate and glutamate metabolism, purine and pyrimidine metabolism, and taurine metabolism were significantly up-regulated In the LNCaP-ADR cells (Fig. 8B).

### Integration of transcriptomic and metabolomic analyses reveals enriched pathway and correlation between genes and metabolites in LNCaP-ADR cells

**Fig. 9:**
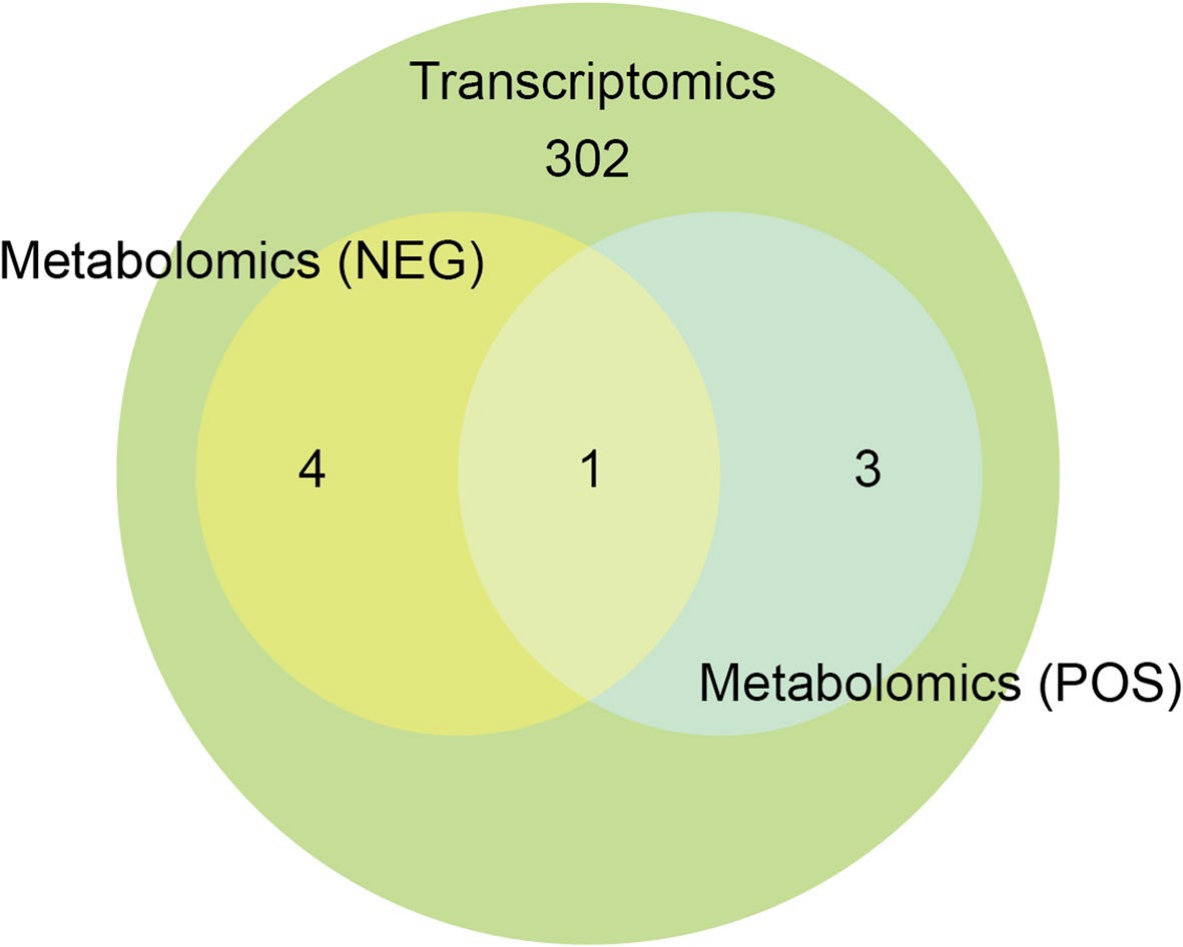
Venn diagram: The mutual KEGG pathway in metabolome using POS and NEG models and transcriptome

The metabolic pathways selected from the metabolomic analysis in both positive ion mode (POS) and negative ion mode (NEG) were compared with the 310 KEGG metabolic pathways identified from the transcriptomic analysis. The Venn diagram (Fig. 9) revealed that only one pathway, Arginine and proline metabolism, was enriched with both differentially expressed genes (DEGs) and differentially expressed metabolites (DEMs).

Further analysis focused on the differential metabolites of Arginine and proline metabolism in both POS and NEG modes. The MS2 score and mean values were compared, resulting in the identification of 17 DEMs (Table 3). Among these, 5 endogenous DEMs (5-Aminopentanoic acid, L-Arginine, L-Glutamic acid, N-Acetyl-L-alanine, and Pyrrole-2-carboxylic acid) were significantly up-regulated in LNCaP-ADR cells compared to parental cells. Additionally, 6 DEGs (*MAOA*, *ALDH3A2*, *ALDH2*, *ARG1*, *CKMT2*, and *CNDP1*) were identified as significantly up-regulated in the LNCaP-ADR cells (LA group) compared to the LNCaP parental cells (L group) (Table 4).

**Table 3:** Identified metabolites in arginine and proline metabolism pathway via combined POS and NEG model analysis

Metabolites	variation trend	MS2 score	MEAN	log2(fc)
5-Aminopentanoic acid	up	1.000	12.911	0.172
L-Arginine	up	0.977	0.579	2.280
L-Glutamic acid	up	0.995	39.837	0.706
N-Acetyl-L-alanine	up	0.998	2.531	0.480
Pyrrole-2-carboxylic acid	up	0.998	1.618	0.864
4-Guanidinobutanoic acid	down	0.983	82.363	-1.949
Argininosuccinic acid	down	0.749	0.330	-0.924
Citrulline	down	0.966	5.967	-0.890
Creatine	down	0.990	140.793	-9.632
Creatinine	down	0.951	27.266	-0.632
Hydroxyproline	down	0.998	7.080	-2.686
L-Aspartic acid	down	0.871	0.050	-0.464
L-Proline	down	1.000	59.569	-0.818
N-Acetylputrescine	down	0.945	11.949	-7.518
Ornithine	down	0.999	1.840	-0.865
Phosphocreatine	down	0.644	0.077	-0.848
N-Acetylornithine	down	0.978	0.669	-2.530

**Table 4:** Differentially expressed genes identified in the arginine and proline metabolism pathway

Gene ID	Gene name	KEGG name	variation trend	log2(fc)
ENSG00000189221	MAOA	K00274	up	1.14
ENSG00000072210	ALDH3A2	NA	up	1.23
ENSG00000111275	ALDH2	NA	up	1.26
ENSG00000118520	ARG1	NA	up	3.56
ENSG00000131730	CKMT2	NA	up	4.35
ENSG00000150656	CNDP1	K05604	up	5.21
ENSG00000166165	CKB	NA	down	-1.69
ENSG00000223572	CKMT1A	K00933	down	-1.11
ENSG00000237289	CKMT1B	NA	down	-1.08

**Fig. 10:**
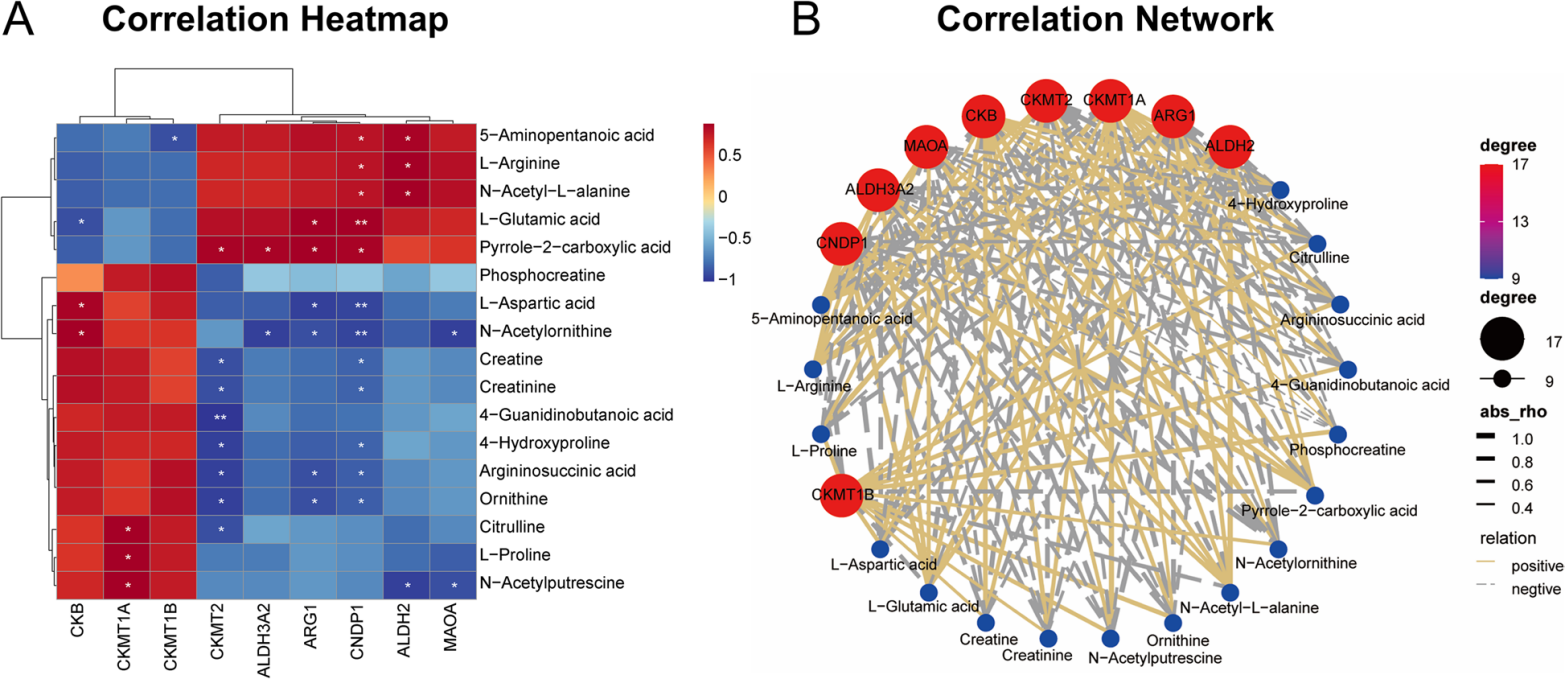
Correlation analysis between differentially expressed metabolites and genes. **A** Heatmap illustrating the correlation between differentially expressed metabolites and genes. The horizontal and vertical axes represent the differentially expressed genes and metabolites used for analysis, respectively. The color of each heatmap block indicates the correlation coefficient obtained through Pearson correlation analysis: red indicates a positive correlation, blue indicates a negative correlation. * denotes a significant correlation between key species and metabolites (*p*-value<0.05), and ** denotes an extremely significant correlation (*p*-value<0.01). **B** Network representation showing the correlation between differentially expressed metabolites (blue nodes) and genes (red nodes). The size of each node reflects its degree of centrality, indicating the number of connections it has with other nodes. The thickness of the lines in the network is determined by the rho value, which indicates the strength of the correlation (absolute value of rho): thicker lines represent stronger correlations (positive or negative), while thinner lines represent weaker correlations. Solid gold lines represent positive correlations, while dashed silver lines represent negative correlations

**Fig. 11:**
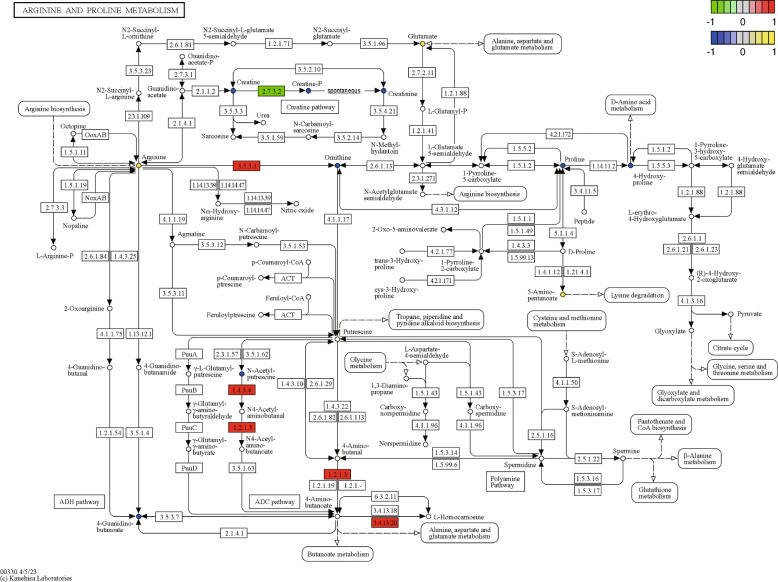
KEGG pathway: Differentially expressed genes and metabolites in arginine and proline metabolism. In the upper right corner of the figure, there are two legends, from top to bottom, for “gene” and “metabolite” respectively. The “gene” legend corresponds to the rectangles in the diagram, while the “metabolite” legend corresponds to the circles. The color intensity in both shapes indicates the expression level, with higher expression depicted as a deeper shade of red

To explore the correlation between the screened DEMs and DEGs, a correlation analysis was performed and visualized using clustering correlation heatmaps and correlation network maps (Fig. 10A, B). The up-regulated DEMs in the LNCaP-ADR cells, such as 5-Aminopentanoic acid, L-Arginine, L-Glutamic acid, N-Acetyl-L-alanine, and Pyrrole-2-carboxylic acid, showed negative correlations with DEGs *CKB*, *CKMT1A*, and *CKMT1B*, while they showed positive correlations with DEGs *CKMT2*, *ALDH3A2*, *ARG1*, *CNDP1*, *ALDH2*, and *MAOA*. Furthermore, 6 DEGs (*CKB*, *CKMT1A*, *CKMT2*, *ARG1*, *CNDP1*, and *ALDH2*) exhibited high correlations with multiple DEMs. The selected up-regulated and down-regulated DEGs and DEMs were identified in KEGG pathways using the tool at https://pathview.uncc.edu and https://www.kegg.jp/pathway/ko00330 [13–15], as shown in Fig. 11.

### Validation of the differentially expressed genes (DEGs) between LNCAP-ADR cells and parental cell

**Fig. 12:**
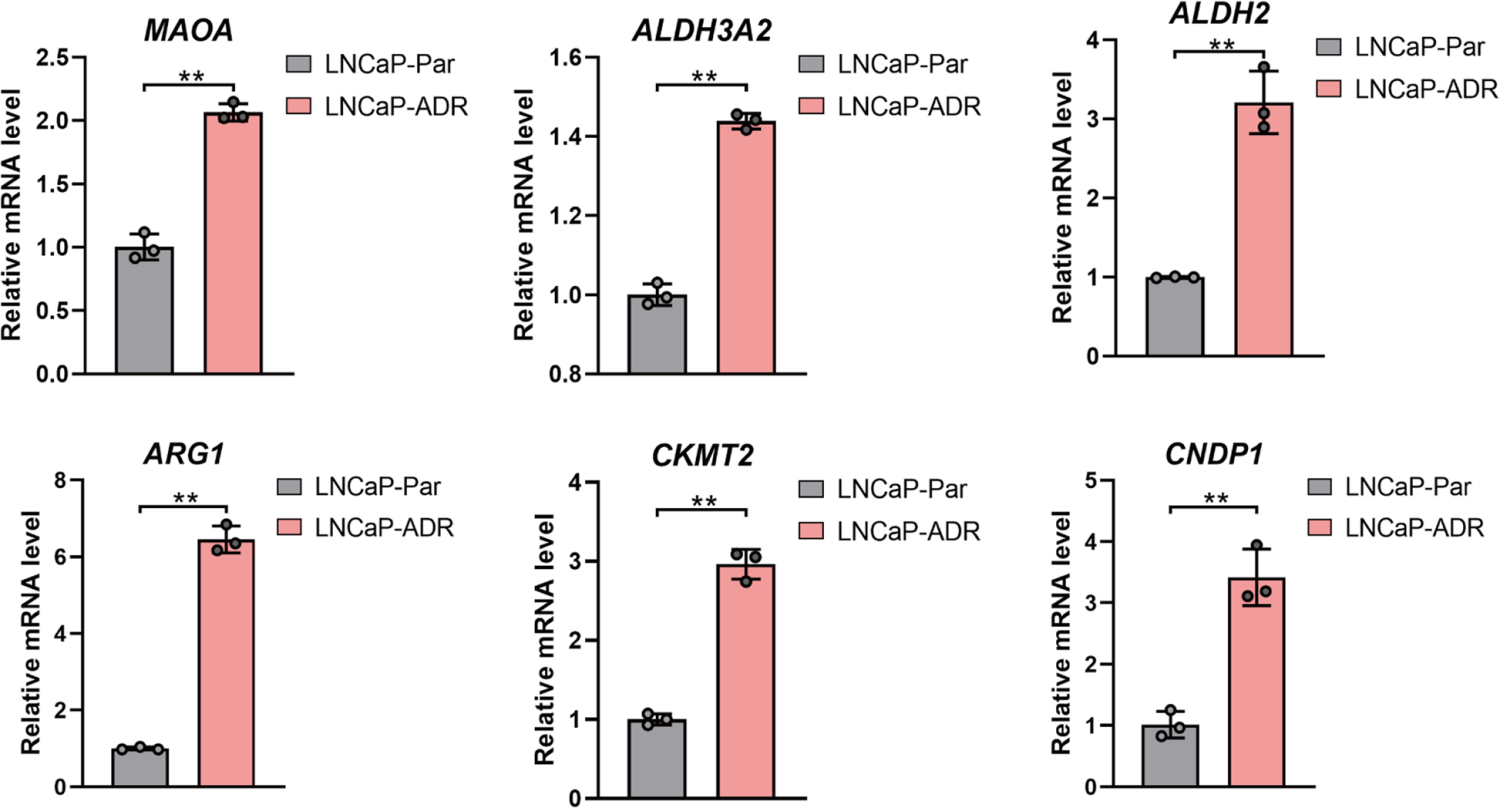
Validating expression levels of 6 DEGs in LNCaP ADR and parental cells. mRNA levels of *MAOA*, *ARG1*, *ALDH2*, *ALDH3A2*, *CKMT2*, and *CNDP1* were detected using QPCR. **P<0.01

To validate DEGs identified through transcriptomics and metabolomics, the six selected genes (*MAOA*, *ALDH3A2*, *ALDH2*, *ARG1*, *CKMT2*, and *CNDP1*) were further confirmed using QPCR. The results demonstrated significant upregulation of mRNA levels of these six DEGs in LNCAP-ADR cells compared to LNCaP parental cells (Fig. 12).

## Discussion

The integration of metabolomic and transcriptomic data in this study offers a comprehensive view of the metabolic alterations associated with LNCaP-ADR cells, uncovering potential key pathways and biomarkers involved in the development of androgen-independent prostate cancer. The identification of the Arginine and proline metabolism pathway as enriched with both DEGs and DEMs highlights its significance in driving the metabolic reprogramming observed in LNCaP-ADR cells.

Within the Arginine and proline metabolism pathway, several metabolites were found to be significantly up-regulated in LNCaP-ADR cells compared to the parental cells. Among these metabolites, 5-Aminopentanoic acid, L-Arginine, L-Glutamic acid, N-Acetyl-L-alanine, and Pyrrole-2-carboxylic acid stood out as potential biomarkers for distinguishing androgen-independent prostate cancer.

Arginine is a fundamental component of protein synthesis and plays a critical role in tumor cell growth. It also serves as an important precursor for polyamines, including putrescine, spermidine, and spermine, which play key roles in cellular proliferation and growth [16]. Moreover, previous reports have indicated that arginine has the ability to directly activate the nutrient-sensing kinase, mammalian target of rapamycin complex 1 (mTORC1), and acts as an epigenetic regulator targeting TEAD4 to modulate oxidative phosphorylation (OXPHOS) in prostate cancer cells [17]. Arginine also participates in the urea cycle, producing nitrogen and energy supply. The urea cycle plays a crucial role in maintaining nitrogen balance in the body and preventing the accumulation of toxic ammonia. In contrast to the toxic effects of excessive ammonia under normal physiological conditions, cancer cells can utilize and recycle ammonia for the synthesis of amino acids and nucleic acids, essential for supporting tumor proliferation and providing the necessary building blocks for proteins and nucleotides [18]. Notably, arginine is also involved in the activation of immunological effector cells and decreasing tumoral immunosurveillance [19]. Previous studies have demonstrated that urine samples or tissues from prostate cancer patients exhibit higher levels of arginine compared to those from patients with benign prostatic hyperplasia (BPH) [20, 21]. Together with our findings, arginine deprivation might represent a novel antimetabolite strategy for the treatment of CRPC.

Five-Aminopentanoic acid, also known as 5-aminovaleric acid, is a degradation product of lysine. It functions as a methylene homologue of γ-aminobutyric acid (GABA) [22]. In advanced prostate cancer, the excessive production of GABA has been reported to directly modulate nuclear androgen receptor signals, thereby contributing to tumorigenesis [23]. Studies have indicated a significant increase in glutamate content in malignant prostate cancer tissue, which is associated with an elevated risk of advanced prostate cancer [24, 25]. Although there are currently no reports on the relationship between Pyrrole-2-carboxylic acid and prostate cancer, a study by Hai Jin et al. demonstrates its high diagnostic capability for metastasis in esophageal squamous cell carcinoma [26]. The increased abundance of these metabolites in LNCaP-ADR cells suggests a rewiring of the metabolic network in the cells, potentially fueling the aggressive phenotype exhibited by these cells.

In LNCaP-ADR cells, we observed upregulation of several genes associated with the arginine and proline metabolic pathways, including *MAOA*, *ALDH3A2*, *ALDH2*, *ARG1*, *CKMT2*, and *CNDP1*. MAOA has been associated with perineural invasion in prostate cancer cells [27]. ALDH3A2 [28–30] and ALDH2 [31–33] are implicated in various cancer types. ARG1 and ARG2 have a positive correlation with LNCaP cell growth [34]. CKMT2 is linked to gastric cancer prognosis and osteosarcoma progression [35, 36]. CNDP1 is involved in carnosine metabolism and reduced plasma levels are associated with poor prognosis in gastrointestinal cancers [37, 38]. The up-regulation of these genes in the LNCaP-ADR cells indicate their association with enhanced migration and invasion capabilities compared to the parental cells. These genes could potentially serve as diagnostic markers for detecting CRPC and supporting clinical treatment.

Furthermore, our GSEA analysis revealed that the LNCaP-ADR cells exhibited enrichment in gene sets associated with inflammatory response, cilium-dependent cell motility, response to endoplasmic reticulum stress, PTEN loss, Src kinases, RB mutations, PI3K-AKT-mTOR signaling, RAS signaling, and angiogenesis. The association between inflammation [39–41], PTEN loss [42], Src kinases [43], RB mutations, PI3K-AKT-mTOR signaling [44], RAS signaling [45], and prostate cancer development have been well established. Cilia play a role in cell motility and fluid transportation [46], and their enrichment in the LNCaP-ADR cells suggests enhanced migration and invasion abilities. Enhanced angiogenesis, mediated by VEGF, is crucial for cancer metastasis, including prostate cancer [47]. These studies supported our findings since CRPC cells exhibit a more aggressive behavior compared to other prostate cancer cells. However, our study has limitations: we solely conducted in vitro validation of the selected DEGs from mRNA level. In our next steps, we intend to conduct further experiments to validate the identified metabolites and genes both in vivo and in vitro, so that to enhance the depth and reliability of our study, and strengthen the overall findings and provide a more robust understanding of the mechanisms involved.

Overall, this integrative analysis of metabolomic and transcriptomic data contributes to our knowledge of the metabolic landscape of androgen-independent prostate cancer. The identified biomarkers and key regulatory genes offer potential avenues for developing targeted therapies and diagnostic tools for more effective management of advanced prostate cancer. Future studies should focus on elucidating the specific roles of these biomolecules and further validating their clinical relevance.

## Data Availability

All untargeted metabolomic data used in this publication have been deposited to the EMBL-EBI MetaboLights database with the identifier MTBLS8377 (liver metabolomics). The complete data set can be accessed at https://www.ebi.ac.uk/metabolights/MTBLS8377. Additional data related to this paper are available upon request from the corresponding author.
